# Association between Age at *Helicobacter pylori* Eradication and the Risk of Gastric Cancer Stratified by Family History of Gastric Cancer: A Nationwide Population-Based Study

**DOI:** 10.3390/cancers15051604

**Published:** 2023-03-04

**Authors:** Yoon Suk Jung, Mai Thi Xuan Tran, Huiyeon Song, Boyoung Park, Chang Mo Moon

**Affiliations:** 1 Division of Gastroenterology, Department of Internal Medicine, Kangbuk Samsung Hospital, Sungkyunkwan University School of Medicine, Seoul 03181, Republic of Korea; 2 Department of Preventive Medicine, Hanyang University College of Medicine, Seoul 04763, Republic of Korea; 3 Department of Epidemiology and Biostatistics, Graduate School of Public Health, Hanyang University, Seoul 04763, Republic of Korea; 4 Department of Internal Medicine, College of Medicine, Ewha Womans University, Seoul 07985, Republic of Korea; 5 Inflammation-Cancer Microenvironment Research Center, College of Medicine, Ewha Womans University, Seoul 07804, Republic of Korea

**Keywords:** gastric cancer, *Helicobacter pylori*, eradication, age

## Abstract

**Simple Summary:**

In this study, in both patients, with and without a family history of GC, young age at *H. pylori* eradication was significantly associated with a reduced risk of GC. The gap in the risk of GC between patients with and without a family history of GC widened as the age at the time of *H. pylori* eradication increased. Our findings suggest that the early treatment of *H. pylori* infection can further maximize the preventive effects of GC.

**Abstract:**

Introduction: This study compares the risk of GC according to age at *H. pylori* eradication, stratified based on the presence of family history of GC using a population-based large cohort. Method: We analyzed individuals who underwent GC screening between 2013 and 2014 and received *H. pylori* eradication therapy before screening. Results: Among 1,888,815 *H. pylori*-treated patients, 2610/294,706 and 9332/1,594,109 patients with and without a family history of GC, respectively, developed GC. After adjusting for confounders, including age at screening, the adjusted hazard ratios (95% confidence intervals) for GC comparison, 70–74, 65–69, 60–64, 55–59, 50–54, 45–49, and <45 years with ≥75 years at *H. pylori* eradication were 0.98 (0.79–1.21), 0.88 (0.74–1.05), 0.76 (0.59–0.99), 0.62 (0.44–0.88), 0.57 (0.36–0.90), 0.38 (0.22–0.66), and 0.34 (0.17–0.67), respectively, among patients with a family history of GC (*p* < 0.001) and 1.01 (0.91–1.13), 0.95 (0.86–1.04), 0.86 (0.75–0.98), 0.67 (0.56–0.81), 0.56 (0.44–0.71), 0.51 (0.38–0.68), and 0.33 (0.23–0.47), respectively, among patients without a family history of GC (*p* < 0.001). Conclusion: In patients with and without a family history of GC, young age at *H. pylori* eradication was significantly associated with a reduced risk of GC, suggesting that the early treatment of *H. pylori* infection can maximize GC prevention.

## 1. Introduction

Gastric cancer (GC) is a serious disease worldwide. With over one million new cases each year, GC is the fifth most commonly diagnosed cancer and the fourth most common cause of cancer death globally [1]. Epidemiologic studies have consistently reported an association between *Helicobacter pylori* (*H. pylori*) infection and GC risk. Based on these results, *H. pylori* is categorized by the International Agency for Cancer Research as a group 1 carcinogen [2].

In the past, there was not much evidence that *H. pylori* eradication reduces the risk of GC, whereas in recent years, this evidence is getting stronger as an increasing number of long-term follow-up randomized controlled trials (RCTs) that prove this have been published [3,4,5]. In a meta-analysis updated in 2020 that assessed seven RCTs involving 8323 healthy individuals, *H. pylori* eradication significantly reduced the incidence of GC by 46% and reduced mortality from GC by 39% [5].

*H. pylori* infection generally occurs in infancy or early childhood and persists for a lifetime if left untreated [6]. Although the effect of *H. pylori* eradication on GC prevention has become clear, it remains questionable as to when the commencement of eradication therapy is more effective at preventing GC. Recent meta-analyses have demonstrated that patients with gastric dysplasia or intestinal metaplasia did not benefit from *H. pylori* eradication in terms of the risk of GC, implying a point of no return in the gastric carcinogenesis cascade associated with *H. pylori* [7,8]. In line with this, the Maastricht V consensus guideline recommends that the risk of GC can be more effectively reduced by *H. pylori* eradication before the development of atrophy and intestinal metaplasia [9]. Based on this theory and the existing literature, it can be expected that the prevention of GC can be further maximized if *H. pylori* eradication is conducted earlier. Surprisingly, however, there have been no studies to prove this seemingly obvious hypothesis. While most previous studies have compared the risk of GC between patients who received *H. pylori* eradication therapy and those who did not, no study has evaluated the effectiveness according to the timing of eradication among *H. pylori*-treated patients.

Therefore, this study aimed to evaluate the association between age at the time of *H. pylori* eradication and the risk of GC by using a population-based large cohort of *H. pylori*-treated patients in Korea. Since the effectiveness of eradication therapy can differ between patients with and without a family history of GC, we conducted analyses after stratifying the study population according to the family history of GC status.

## 2. Materials and Methods

### 2.1. Data Source and Identification of the Study Population

This was a retrospective cohort study of participants who underwent GC screening. Data were obtained from a customized dataset of the National Health Insurance Service-National Health Information Database (NHIS-NHID) in Korea. Participants with a family history of any cancer and an age- and sex-matched random sample of participants without a family history of cancer were included. The NHIS is a compulsory health insurance system that covers all forms of medical services of the entire Korean population [10]. All Koreans aged ≥40 years are provided biennial esophagogastroduodenoscopy screening under the National Cancer Screening Programme [11].

**Figure 1:**
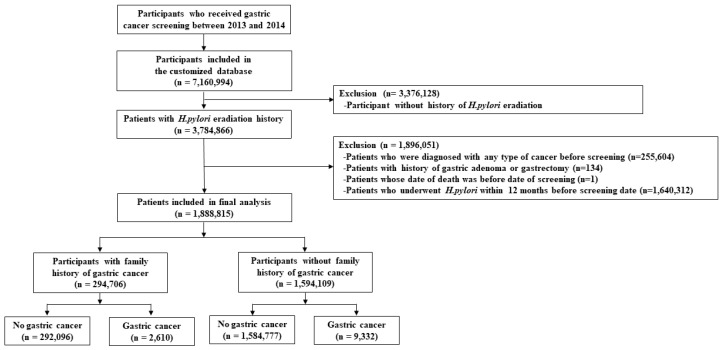
Flowchart for the selection of the study population. *H. pylori*, *Helicobacter pylori*.

Our initial database included claims data from 2011 to 2018. Since previous prevalent GC or other types of cases may confound the association for incidence, we used a washout period of 2 years from 2011 to 2012 and excluded subjects who had history of hospital visits due to any type of cancer during the period. Accordingly, we analyzed participants who had undergone GC screening between 1 January 2013, and 31 December 2014, and had received *H. pylori* eradication therapy before screening. Our target study cohort included 7,160,994 adults aged ≥40 years who underwent GC screening between 2013 and 2014. Next, we selected 3,784,866 patients who had received *H. pylori* eradication therapy before screening. Of these, 255,604 and 134 patients were excluded because of a history of any type of cancer including GC and a history of gastric adenoma or gastrectomy during the washout period. One patient was excluded because the date of death was recorded prior to the screening date. In addition, 1,640,312 patients who received *H. pylori* eradication therapy within 12 months before the screening date were excluded because 12 months or less may not be sufficient to evaluate the effect of the therapy. Finally, 1,888,815 patients were included in the analysis. Since a family history of GC is a well-known high-risk factor for GC [12,13], we analyzed the association between age at *H. pylori* eradication and GC risk under stratification based on family history of GC. Among them, 294,706 patients had a family history of GC, while 1,594,109 patients did not (Figure 1). 

Approval for this study was granted by the Institutional Review Board (IRB) of Ewha Womans University Mokdong Hospital (IRB No. 2020-08-030). Informed consent was waived because all screened populations agreed to transfer their screening results to NHIS-NHID, and the NHIS database was constructed after the data was anonymized.

### 2.2. Main Outcome and Definition of Variables

This study analyzed the association between age at the time of *H. pylori* eradication and the risk of GC as a top priority. In addition, we stratified these analyses based on family history of GC. We considered that a family history of GC was a powerful risk factor for GC [12,13], which can strongly impact the main research topic (the association between age at *H. pylori* eradication and GC risk). Thus, we analyzed the association between age at *H. pylori* eradication and GC risk under stratification based on family history of GC, rather than correcting it as a confounding factor in multivariate analyses.

The main outcome of this study was incident GC. GC was defined as a combination of the International Classification of Disease (ICD)-10 code for GC (C16) and the rare and intractable disease (RID) code for cancer. The RID code is related to the cost sharing of the out-of-pocket money for diseases with a high financial burden in Korea, including cancer, and the use of this code in combination with ICD-10 codes increases the accuracy of GC ascertainment [14]. A Korean study investigated the validity of cancer diagnosis based on the NHIS database vs. the National Cancer Registry database in Korea [14]. In this study, the sensitivity and positive predictive value (PPV) of the primary diagnosis-based definition of GC were 96.0% (95% confidence interval [95% CI] 96.0–96.1) and 94.1% (95% CI 94.0–94.2), respectively, and the sensitivity and PPV of the RID-based definition of GC were 95.7% (95% CI 95.7–95.8) and 93.9% (95% CI 93.8–94.0), respectively [14].

Gastric adenoma was defined as those diagnosed with ICD-10 codes D00.2 or D13.1 and a history of endoscopic resection or gastrectomy within 6 months after the above-mentioned diagnosis. To ascertain the newly diagnosed GC cases, we excluded participants diagnosed with any cancer and participants who had a history of gastrectomy or gastric adenoma during the washout period. All participants were followed until the date of GC, date of death, or 31 December 2018, whichever came first.

We defined *H. pylori* eradication therapy as the simultaneous prescription of a proton pump inhibitor (PPI), clarithromycin, and amoxicillin or a PPI, tetracycline, and metronidazole within 7 days from the date of first prescription of any of the above-mentioned drugs from 1 January 2011 to 12 months before the date of GC screening. This definition includes both first-line and second-line eradication therapies approved in Korea, as well as combination and sequential therapies [15]. In our analysis, age at *H. pylori* eradication was categorized into the following 5-year age groups: ≥75, 70–74, 65–69, 60–64, 55–59, 50–54, 45–49, and <45 years. 

Information on the family history of cancer (yes/no), smoking habits (never, former, and current smokers), alcohol consumption (0 or 1/week and ≥2/week), weekly physical activity (no and yes [≥1/week]), and comorbidity status (yes/no) was obtained through a self-administered questionnaire. All participants reported whether they had any first-degree relatives (FDRs), including parents, siblings, and children, who were previously diagnosed with cancer. Based on this information, a family history of GC was defined as GC with ≥1 FDR, while no family history of GC was defined as the absence of a family history of any type of cancer. Comorbidity status was defined as having at least one of the following: hypertension, diabetes mellitus, dyslipidemia, ischemic heart disease, or stroke, based on the questionnaire. Height and weight were measured during screening and used to calculate body mass index (BMI) as weight in kilograms divided by height in meters squared. Obesity status was defined as a BMI ≥ 25 kg/m^2^.

To infer the indications for *H. pylori* eradication, the following diagnostic codes (ICD-10 codes) at the time of prescribing eradication therapy were identified: K25 for gastric ulcer, K26 for duodenal ulcer, “both K25 and K26” or “K27” for gastric and duodenal ulcer, and C88.4 for gastric mucosa-associated lymphoid tissue lymphoma.

### 2.3. Statistical Analysis

Baseline characteristics between participants with and without a family history of GC were compared using Student’s t-test for continuous variables and the χ^2^ test for categorical variables. The Grey test was conducted for statistical comparisons of cumulative incidence functions among the age groups of *H. pylori* eradication [16]. Crude and adjusted Cox proportional hazards regression analysis was conducted to calculate the hazard ratios (HR) and 95% CIs to quantify the risk of GC. For the adjusted analysis, age at screening, sex, obesity status, smoking habit, alcohol consumption, physical activity, and comorbidity status were adjusted. All analyses for the association between age at *H. pylori* eradication and GC risk were stratified based on the status of family history of GC. Kaplan–Meier curves were used to test the proportional hazards assumptions and to identify parallel lines of the log-log survival distribution function. All *p*-values were two-sided with a type I error (α < 0.05) that is considered statistically significant. Statistical analyses were performed using SAS statistical software (version 9.4, SAS Institute).

## 3. Results

### 3.1. Baseline Characteristics of the Study Population

Among the 1,888,815 subjects selected by inclusion and exclusion criteria, 294,706 (15.6%) had a family history of GC. The baseline characteristics of the patients with and without a family history of GC are shown in Table 1. The mean follow-up time in the study population was 4.77 years, with a maximum of 6 (median, 4.75; interquartile range [IQR], 4.28–5.22) years. The mean age of the patients was 57.1 ± 9.8 years, and 61.1% were men.

### 3.2. Risk of GC According to Age at H. pylori Eradication in Patients with a Family History

The mean follow-up period was 4.78 years, with a maximum of 6 (median, 4.76; IQR, 4.29–5.25) years in patients with a family history of GC. During the follow-up period, among 294,706 patients with a family history of GC, 2610 (0.9%) developed GC.

Table 2 shows the risk of GC in relation to age at *H. pylori* eradication in patients with a family history of GC.

**Figure 2:**
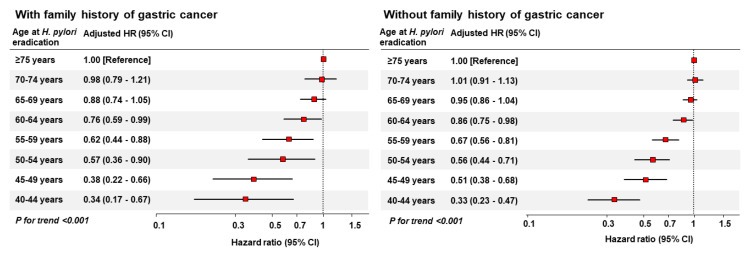
Risk of gastric cancer according to age at *H. pylori* eradication in patients with and without a family history of gastric cancer. *H. pylori*, *Helicobacter pylori*; *HR*, *hazard ratio*; *CI*, *confidence interval*.

In the crude model, decreasing age at *H. pylori* eradication was significantly associated with a decreased risk of GC. Even after adjusting for age at screening, sex, obesity, smoking habit, alcohol consumption, physical activity, and comorbidities, younger age at *H. pylori* eradication was significantly associated with a decreased risk of GC (*p* trend < 0.001). The adjusted HRs (95% CIs) for GC comparing 70–74, 65–69, 60–64, 55–59, 50–54, 45–49, and <45 years with ≥75 years at *H. pylori* eradication were 0.98 (0.79–1.21), 0.88 (0.74–1.05), 0.76 (0.59–0.99), 0.62 (0.44–0.88), 0.57 (0.36–0.90), 0.38 (0.22–0.66), and 0.34 (0.17–0.67), respectively, which are illustrated in Figure 2. Age at screening, male sex, and former or current smoking status were also identified as independent risk factors for GC.

### 3.3. Risk of GC According to Age at H. pylori Eradication in Patients without a Family History

The mean follow-up period was 4.76 years, with a maximum of 6 (median, 4.75; IQR 4.27–5.22) years in patients without a family history of GC. During the follow-up period, among 1,594,109 patients without a family history of GC, 9332 (0.6%) developed GC. Table 3 shows the risk of GC in relation to age at *H. pylori* eradication in patients without a family history of GC.

The results were similar to those of patients without a family history of GC. Even after adjusting for confounders, including age at screening, a decrease in the age at *H. pylori* eradication was significantly associated with a decrease in the risk of GC even in patients without a family history of GC (*p* trend < 0.001). The adjusted HRs (95% CIs) for GC comparing 70–74, 65–69, 60–64, 55–59, 50–54, 45–49, and <45 years with ≥75 years at *H. pylori* eradication were 1.01 (0.91–1.13), 0.95 (0.86–1.04), 0.86 (0.75–0.98), 0.67 (0.56–0.81), 0.56 (0.44–0.71), 0.51 (0.38–0.68), and 0.33 (0.23–0.47), respectively (Figure 2). Additionally, age at screening, male sex, former or current smoking, and alcohol consumption were found to be independent risk factors for GC, whereas physical activity (≥one time/week) was an independent protective factor against GC. 

During the study period, most patients received standard triple therapy. The proportions of patients who received standard triple therapy and sequential/combination therapy were 93.1% (n = 1,757,599) and 6.8% (n = 127,566), respectively. We compared family history status, age at *H. pylori* eradication, and the development of GC between patients treated with standard triple therapy and those with sequential/concomitant therapy (Table 4). After adjusting for confounders, patients who received sequential or concomitant therapy showed a 1.62-fold increased risk of GC compared with those who received standard triple therapy.

In this study, we also analyzed the association between age at *H. pylori* eradication and the risk of gastric adenoma (Table 5). After adjusting for confounders, a decrease in the age at *H. pylori* eradication was significantly associated with a decrease in the risk of gastric adenoma in both patients with and without a family history of GC (*p* trend < 0.001).

To adjust for the influence of age itself on GC development, we analyzed the risk of GC according to age at *H. pylori* eradication when all participants within an age group were followed up until all reached a comparable age (Table 6). In each age group, the oldest one was used as reference and was compared with risk in other groups. As a result, we observed a significant decrease in the HRs for GC in the younger age groups, especially in the age groups 40–44, 50–54, 55–59, and 60–64. These findings can support our hypothesis that an earlier age at *H. pylori* eradication decreased the risk of GC. 

## 4. Discussion

In this population-based study, we found that in both patients with and without a family history of GC, the younger the patient’s age at the time of *H. pylori* eradication, the lower the risk of GC. Because age itself is an important risk factor for GC, age at screening was adjusted, but our results were, nevertheless, significantly maintained. Our findings suggest that GC prevention can be further maximized by eradicating *H. pylori* in the early stages of infection. To the best of our knowledge, this is the first study to identify the effect of the timing of *H. pylori* eradication on GC risk among *H. pylori*-treated patients.

Treatment of *H. pylori* infection has consistently been shown to reduce the risk of GC in recent meta-analyses [3,4,5]. However, since *H. pylori* infection is usually acquired early in life and persists for a lifetime, the question remains as to when treatment is the most optimal. Given that *H. pylori* infection leads to a sequence of events known as Correa’s cascade, a stepwise progression from chronic gastric inflammation, atrophy, intestinal metaplasia, dysplasia, to GC [17], it can be theoretically expected that blocking this series of processes at an early stage will increase the effectiveness of GC prevention. Several studies have supported this hypothesis. Two meta-analyses have revealed that the role of *H. pylori* eradication therapy after the development of pre-neoplastic changes, such as intestinal metaplasia or dysplasia is unclear [7,8]. A meta-analysis also highlighted that severe atrophy and intestinal metaplasia may increase the risk of metachronous GC after endoscopic resection in *H. pylori* eradicated patients [18]. In addition, some studies have reported that the effect of *H. pylori* eradication is affected by the degree of atrophic gastritis. Therefore, the effect on GC prevention is higher in patients with mild atrophic gastritis than in patients with extensive or severe atrophic gastritis [19,20]. These results suggest that there is a point of no return in the gastric carcinogenesis cascade associated with *H. pylori*; thus, *H. pylori* eradication should be performed before atrophy, or intestinal metaplasia occurs.

To date, no studies have compared the risk of GC according to age at the time of eradication among *H. pylori*-infected patients. Moreover, only two studies have evaluated the appropriate timing for *H. pylori* eradication. A large cohort study of 371,813 patients diagnosed with *H. pylori* infection in the United States showed a 13% increase in the risk of GC for each 5-year increase in age at *H. pylori* diagnosis (sub-HR 1.13; 95% CI 1.11–1.15) [21]. Although this study analyzed the age at which *H. pylori* infection was diagnosed, not the age at which *H. pylori* infection was treated, it suggests that diagnosing and treating *H. pylori* infection at a younger age may be beneficial in preventing GC. Another study of 73,237 patients in Hong Kong treated for *H. pylori* infection reported that *H. pylori*-treated patients aged <40 and 40–59 years did not differ in GC incidence compared to the general population, whereas *H. pylori*-treated patients aged ≥60 years showed an 18% reduction in GC incidence compared to the general population (standardized incidence ratio 0.82; 95% CI 0.69–0.97) [22]. However, a limitation of this study was that the comparison group was the general population, which included both *H. pylori*-infected and uninfected individuals rather than those who did not receive eradication therapy. Therefore, it is not possible to determine whether eradication therapy had a greater effect on the prevention of GC in the elderly than in the young population. This is because the most common age for GC diagnosis is over 60 [23], and most patients aged <40 and 40–59 years have not yet been followed up to the age at which GC is most prevalent. If the follow-up period was extended sufficiently, it would have been possible to confirm the effect of reducing the risk of GC even in individuals aged <60. Rather, it is reasonable to interpret these results as being able to prevent GC through *H. pylori* eradication, even for those aged >60.

Although our results revealed that treating *H. pylori* infection at a younger age can increase the effectiveness of GC prevention, there is still insufficient evidence as to when *H. pylori* eradication treatment should be started. If *H. pylori* eradication is performed in childhood, the chance of re-infection may be high, and antibiotic resistance may increase due to early exposure to antibiotics. Therefore, we would like to recommend *H. pylori* eradication in early adulthood after adolescence.

Another valuable finding of our study was that younger age at *H. pylori* eradication was associated with a lower risk of gastric adenoma. These findings support that *H. pylori* eradication may have a role in GC prevention by influencing the early stages of adenoma initiation and development in the gastric carcinogenesis, and further clearly suggest the benefit of early eradication.

Another finding of our study was that, even if *H. pylori* eradication treatment was administered, the gap in the risk of GC between patients with and without a family history of GC widened as the age at the time of *H. pylori* eradication increased. These findings emphasize the importance of early eradication for patients with a family history, a high-risk group for GC.

Our study had several limitations. First, the results of *H. pylori* eradication were not available in the NHIS-NHID. As this study was conducted retrospectively, a follow-up test to confirm the effectiveness of *H. pylori* eradication was not performed for all the study subjects. Additionally, information regarding whether the test was conducted is available, but information concerning the results is not available due to the nature of our cohort data. However, our results may have been more significant if we only evaluated patients who were successful in eradicating *H. pylori*, as shown in previous studies [21,24]. In addition, there was limited information regarding *H. pylori* infection, exposure time, and recurrence in our current database. Therefore, it was difficult to analyze the effect of this confounding factor on the outcome (risk of GC) and to control its influence as a confounding variable. Especially, the duration between *H. pylori* infection and eradication therapy may be a major factor in the development of dysplasia, which may serve as a confounder in our results. Second, the effect of *H. pylori* eradication in patients in their 20s or 30s could not be evaluated. Since the national GC screening program was provided to individuals aged ≥40 years and we were able to extract the prescription drug code from 2 years before the screening, the age at which *H. pylori* eradication therapy was performed could be analyzed from the age of 38. Third, there was limited data to analyze the efficacy of the type of each *H. pylori* eradication regimen in reducing GC risk. These results may be influenced by the proportion of each regimen used as the first-line therapy. However, in previously published Korean guidelines (2013) [25], sequential or concomitant therapy was not recommended as a first-line therapy because of the limited number of studies on them at that time. Since our study period coincided with the time when the 2013 guideline was implemented, almost all participants in our study were treated with standard triple therapy as a first-line regimen. Fourth, the follow-up observation period for GC occurrence was short. If we had been able to follow up younger adults under the age of 40 who received *H. pylori* eradication treatment for a longer period, the effect of early eradication on GC prevention might have been more clearly identified. Fifth, this study included gastric or duodenal ulcers as indications for *H. pylori* eradication treatment. However, it cannot be attributed to *H. pylori* infection itself, as a number of causes can cause gastric or duodenal ulcers. Lastly, although *H. pylori* infection is more related to non-cardiac GC [26], an analysis according to GC location was not performed because such information was not available in the NHIS-NHID.

## 5. Conclusions

In conclusion, a younger age at *H. pylori* eradication was significantly associated with a lower risk of GC in patients with and without a family history of GC. The gap in the incidence of GC between patients with and without a family history of GC widened as age at the time of eradication of *H. pylori* increased. Our results strongly support screening for *H. pylori* infection in early adulthood and the administration of early eradication therapy when the infection is confirmed, to maximize GC prevention. To verify our results, further prospective studies with long-term follow-up and detailed data on *H. pylori* infection and its eradication are needed.

## Figures and Tables

**Table 1:** Baseline characteristics of all *H. pylori*-treated patients.

Characteristics	Family History of GC (n = 294,706)	No Family History of GC (n = 1,594,109)	*p*-Value
Age at screening (years)	57.1 ± 9.7	57.1 ± 9.8	<0.001
Age group at screening			
40–49 years	66,376 (22.5)	367,285 (23.0)	<0.001
50–59 years	109,451 (37.1)	579,176 (36.3)	
60–69 years	82,100 (27.9)	440,026 (27.6)	
≥70 years	36,779 (12.5)	207,622 (13.0)	
Sex			
Female	123,644 (42.0)	613,556 (38.5)	<0.001
Male	171,062 (58.0)	980,553 (61.5)	
Obesity (BMI ≥ 25 kg/m^2^)			
No	190,779 (64.7)	1,021,777 (64.1)	<0.001
Yes	103,902 (35.3)	572,168 (35.9)	
Unknown	25 (0.0)	164 (0.0)	
Smoking habit			
Never	195,926 (66.5)	1,115,474 (70.0)	<0.001
Former	51,370 (17.4)	232,825 (14.6)	
Current	47,309 (16.1)	245,282 (15.4)	
Unknown	101 (0.0)	528 (0.0)	
Alcohol consumption			
0 or 1 time per week	227,576 (77.2)	1,252,957 (78.6)	<0.001
≥2 times per week	66,897 (22.7)	340,069 (21.3)	
Unknown	233 (0.1)	1083 (0.1)	
Physical activity			
No	66,836 (22.7)	394,668 (24.8)	<0.001
Yes (≥1 time per week)	227,552 (77.2)	1,197,894 (75.2)	
Unknown	318 (0.1)	1547 (0.1)	
Comorbidities			
Hypertension	80,762 (27.4)	462,299 (29.0)	<0.001
Diabetes mellitus	30,353 (10.3)	170,240 (10.7)	
Dyslipidemia	23,646 (8.0)	120,842 (7.6)	
Ischemic heart disease	11,569 (3.9)	61,131 (3.8)	
Stroke	3656 (1.2)	19,605 (1.2)	
Comorbidity status (yes)	108,743 (36.9)	606,308 (38.0)	<0.001
Indication for *H. pylori* eradication			
Gastric ulcer	61,147 (20.7)	320,248 (20.1)	<0.001
Duodenal ulcer	15,229 (5.2)	79,547 (5.0)	
Gastric and duodenal ulcer ^a^	13,469 (4.6)	74,606 (4.7)	
Gastric MALT lymphoma	4 (0.0)	3 (0.0)	
*H. pylori*-associated gastritis	204,857 (69.5)	1,119,705 (70.2)	

GC, gastric cancer; BMI, body mass index; *H. pylori*, *Helicobacter pylori;* MALT, mucosa-associated lymphoid tissue. Values are presented as mean ± standard deviation or number (%). ^a^ Gastric ulcer and duodenal ulcer/ gastroduodenal ulcer unspecified.

**Table 2:** Risk of gastric cancer according to age at *H. pylori* eradication in patients with a family history of gastric cancer.

Variables	N	Person–Years	Incidence (95% CI) ^a^	Crude HR (95% CI)	Adjusted HR (95% CI)
Age at *H. pylori* eradication					
≥75 years	214	45,998	4.65 (4.03–5.28)	1 (Reference)	1 (Reference)
70–74 years	287	69,429	4.13 (3.66–4.61)	0.89 (0.75–1.06)	0.98 (0.79–1.21)
65–69 years	553	172,833	3.20 (2.93–3.47)	0.69 (0.59–0.81)	0.88 (0.74–1.05)
60–64 years	396	162,193	2.44 (2.20–2.68)	0.52 (0.44–0.62)	0.76 (0.59–0.99)
55–59 years	544	314,374	1.73 (1.59–1.88)	0.37 (0.32–0.44)	0.62 (0.44–0.88)
50–54 years	311	225,437	1.38 (1.23–1.53)	0.30 (0.25–0.35)	0.57 (0.36–0.90)
45–49 years	157	187,873	0.84 (0.70–0.97)	0.18 (0.15–0.22)	0.38 (0.22–0.66)
40–44 years	148	229,125	0.65 (0.54–0.75)	0.14 (0.11–0.17)	0.34 (0.17–0.67)
*p* for trend				<0.001	<0.001
Age at screening, per year				1.06 (1.06–1.06)	1.03 (1.01–1.05)
Sex					
Female	970	822,475	1.18 (1.11–1.25)	1 (Reference)	1 (Reference)
Male	1640	584,787	2.80 (2.67–2.94)	2.37 (2.19–2.57)	2.09 (1.88–2.34)
Obesity (BMI ≥ 25 kg/m^2^)					
No	1681	910,886	1.85 (1.76–1.93)	1 (Reference)	1 (Reference)
Yes	928	496,257	1.87 (1.75–1.99)	1.01 (0.94–1.10)	0.95 (0.87–1.03)
Smoking habit					
Never	1348	940,476	1.43 (1.36–1.51)	1 (Reference)	1 (Reference)
Former	700	243,307	2.88 (2.66–3.09)	2.00 (1.83–2.19)	1.17 (1.04–1.31)
Current	561	222,999	2.52 (2.31– 2.72)	1.75 (1.59–1.93)	1.38 (1.22–1.56)
Alcohol consumption					
0 or 1 time/week	1869	1,089,055	1.72 (1.64–1.79)	1 (Reference)	1 (Reference)
≥2 times/week	740	317,127	2.33 (2.17–2.50)	1.36 (1.25–1.48)	1.08 (0.98–1.19)
Physical activity					
No	603	320,047	1.88 (1.73–2.03)	1 (Reference)	1 (Reference)
Yes (≥1 time/week)	2000	1,085,671	1.84 (1.76–1.92)	0.98 (0.89–1.07)	0.97 (0.88–1.06)
Comorbidity status ^b^					
No	1353	887,775	1.52 (1.44–1.61)	1 (Reference)	1 (Reference)
Yes	1257	519,487	2.42 (2.29–2.55)	1.59 (1.47–1.72)	1.03 (0.95–1.12)

*H. pylori*, *Helicobacter pylori*; CI, confidence interval; HR, hazard ratio; BMI, body mass index. ^a^ Incidence rate was calculated as cases per 1000 person–years. ^b^ Comorbidity status was defined as having at least one of hypertension, diabetes mellitus, dyslipidemia, ischemic heart disease, and stroke.

**Table 3:** Risk of gastric cancer according to age at *H. pylori* eradication in patients without a family history of gastric cancer.

Variables	N	Person–Years	Incidence (95% CI) ^a^	Crude HR (95% CI)	Adjusted HR (95% CI)
Age at *H. pylori* eradication					
≥75 years	827	262,139	3.15 (2.94–3.37)	1 (Reference)	1 (Reference)
70–74 years	1027	390,647	2.63 (2.47–2.79)	0.84 (0.76–0.92)	1.01 (0.91–1.13)
65–69 years	2039	953,595	2.14 (2.05–2.23)	0.68 (0.63–0.74)	0.95 (0.86–1.04)
60–64 years	1462	861,892	1.70 (1.61–1.78)	0.54 (0.49–0.59)	0.86 (0.75–0.98)
55–59 years	1884	1,669,636	1.13 (1.08–1.18)	0.36 (0.33–0.39)	0.67 (0.56–0.81)
50–54 years	953	1,181,730	0.81 (0.76–0.86)	0.26 (0.23–0.28)	0.56 (0.44–0.71)
45–49 years	663	988,615	0.67 (0.62–0.72)	0.21 (0.19–0.24)	0.51 (0.38–0.68)
40–44 years	477	1,285,235	0.37 (0.34–0.40)	0.12 (0.11–0.13)	0.33 (0.23–0.47)
*p* for trend				<0.001	<0.001
Age at screening, per year				1.06 (1.06–1.06)	1.03 (1.02–1.04)
Sex					
Female	3539	4,700,031	0.75 (0.73–0.78)	1 (Reference)	1 (Reference)
Male	5793	2,893,458	2.00 (1.95–2.05)	2.65 (2.54–2.76)	2.35 (2.23–2.49)
Obesity (BMI ≥ 25 kg/m^2^)					
No	5800	4,865,695	1.19 (1.16–1.22)	1 (Reference)	1 (Reference)
Yes	3531	2,727,062	1.29 (1.25–1.34)	1.09 (1.04–1.13)	1.02 (0.98–1.07)
Smoking habit					
Never	5094	5,336,735	0.95 (0.93–0.98)	1 (Reference)	1 (Reference)
Former	2140	1,099,546	1.95 (1.86–2.03)	2.03 (1.93–2.14)	1.12 (1.05–1.19)
Current	2094	1,154,625	1.81 (1.74–1.89)	1.90 (1.80–1.99)	1.39 (1.31–1.48)
Alcohol consumption					
0 or 1 time/week	6741	5,979,969	1.13 (1.10–1.15)	1 (Reference)	1 (Reference)
≥2 times/week	2589	1,608,489	1.61 (1.55–1.67)	1.43 (1.36–1.49)	1.06 (1.01–1.12)
Physical activity					
No	2496	1,884,265	1.32 (1.27–1.38)	1 (Reference)	1 (Reference)
Yes (≥1 time/week)	6827	5,701,664	1.20 (1.17–1.23)	0.90 (0.86–0.94)	0.92 (0.88–0.97)
Comorbidity status ^b^					
No	4699	4,704,372	1.00 (0.97–1.03)	1 (Reference)	1 (Reference)
Yes	4633	2,889,117	1.60 (1.56–1.65)	1.61 (1.54–1.67)	1.02 (0.98–1.06)

*H. pylori*, *Helicobacter pylori*; CI, confidence interval; HR, hazard ratio; BMI, body mass index; ^a^ Incidence rate was calculated as cases per 1000 person–years. ^b^ Comorbidity status was defined as having at least one of hypertension, diabetes mellitus, dyslipidemia, ischemic heart disease, and stroke.

**Table 4:** Distribution of *H. pylori* eradication regimens according to family history, age group and cancer development status.

Factors		*H. pylori* Eradication Regimen				
		Standard Triple Therapy ^a^		Sequential or Concomitant Therapy ^b^		*p*-Value
		**n**	**%**	**n**	**%**	
		**n = 1,757,599**		**n = 127,566**		
**Characteristics**						
Family history status						
	No	1,483,863	84.4	107,309	84.1	0.004
	Yes	273,736	15.6	20,257	15.9	
Age group						
	≥75 years	63,261	3.6	2437	1.9	<0.001
	70–74 years	92,193	5.2	3841	3.0	
	65–69 years	223,100	12.7	10,609	8.3	
	60–64 years	202,709	11.5	11,110	8.7	
	55–59 years	387,240	22.0	25,883	20.3	
	50–54 years	271,491	15.4	22,461	17.6	
	45–49 years	227,723	13.0	21,077	16.5	
	40–44 years	289,882	16.5	30,148	23.6	
Cancer development						
	No	1,746,513	99.4	126,745	99.4	0.312
	Yes	11,086	0.6	821	0.6	
**Risk of gastric cancer development**						
Crude HR		Reference		**1.22 (1.13–1.31)**		
Adjusted HR ^c^		Reference		**1.62 (1.16–2.25)**		

^a^ Standard triple therapy was defined as the concomitant prescription of proton pump inhibitors (PPI), clarithromycin, and amoxicillin. ^b^ Sequential therapy was defined as the concomitant prescription of PPI and amoxicillin for 5 days followed by PPI, clarithromycin, and metronidazole for 5 days. Concomitant therapy was defined as the concomitant prescription of PPI, clarithromycin, amoxicillin, and metronidazole. ^c^ Hazard ratios from the multivariate model were adjusted for age at screening, sex, obesity, smoking habit, alcohol consumption, physical activity, and comorbidities.

**Table 5:** Risk of gastric adenoma according to the age at *H. pylori* eradication in total study population and the patients with and without family history of gastric cancer.

Variables	N	Crude HR (95% CI)	Adjusted HR ^a^ (95% CI)
**Total**			
Age at *H. pylori* eradication	n = 6899		
≥60 years	3756	1 (Reference)	1 (Reference)
55–59 years	1292	0.64 (0.60–0.68)	0.72 (0.66–0.78)
50–54 years	1130	0.47 (0.44–0.51)	0.56 (0.50–0.63)
45–49 years	439	0.29 (0.26–0.32)	0.35 (0.30–0.41)
40–44 years	282	0.14 (0.13–0.16)	0.18 (0.15–0.22)
*p* for trend		<0.001	<0.001
**With family history**			
Age at *H. pylori* eradication	n = 1450		
≥60 years	775	1 (Reference)	1 (Reference)
55–59 years	264	0.62 (0.54–0.71)	0.68 (0.56–0.82)
50–54 years	251	0.49 (0.43–0.57)	0.57 (0.45–0.73)
45–49 years	96	0.29 (0.24–0.36)	0.35 (0.25–0.49)
40–44 years	64	0.16 (0.12–0.21)	0.20 (0.13–0.31)
*p* for trend		<0.001	<0.001
**Without family history**			
Age at *H. pylori* eradication	n = 5449		
≥60 years	2981	1 (Reference)	1 (Reference)
55–59 years	1028	0.65 (0.60–0.69)	0.73 (0.66–0.80)
50–54 years	879	0.47 (0.43–0.50)	0.56 (0.49–0.63)
45–49 years	343	0.28 (0.25–0.32)	0.35 (0.30–0.42)
40–44 years	218	0.14 (0.12–0.16)	0.18 (0.15–0.22)
*p* for trend		<0.001	<0.001

*H. pylori*, *Helicobacter pylori*; HR, hazard ratio; CI, confidence interval; ^a^ Hazard ratios from multivariate model were adjusted for age at screening, sex, obesity, smoking habit, alcohol consumption, physical activity, and comorbidities. Regression model for total population was additionally adjusted for family history of gastric cancer status.

**Table 6:** Risk of gastric cancer according to age at *H. pylori* eradication by age group.

Age at *H. pylori* Eradication ^a^		Total Population		With Family History		Without Family History	
		HR (95% CI) ^b^	*p* ^c^	HR (95% CI) ^b^	*p* ^c^	HR (95% CI) ^b^	*p* ^c^
40–44	Age 44→45	Ref.	<0.001	Ref.	0.353	Ref.	<0.001
	Age 43→45	0.98 (0.75–1.29)		1.24 (0.71–2.16)		0.91 (0.67–1.25)	
	Age 42→45	0.86 (0.67–1.11)		0.83 (0.48–1.43)		0.87 (0.66–1.15)	
	Age 41→45	0.78 (0.58–1.06)		1.16 (0.64–2.09)		0.68 (0.47–0.97)	
	Age 40→45	0.70 (0.56–0.86)		0.85 (0.54–1.34)		0.66 (0.52–0.83)	
45–49	Age 49→50	Ref.	0.308	Ref.	0.693	Ref.	0.345
	Age 48→50	1.09 (0.89–1.33)		1.10 (0.68–1.75)		1.09 (0.87–1.36)	
	Age 47→50	1.15 (0.92–1.44)		1.14 (0.68–1.92)		1.15 (0.89–1.48)	
	Age 46→50	0.88 (0.71–1.10)		0.96 (0.59–1.57)		0.86 (0.68–1.10)	
	Age 45→50	0.96 (0.76–1.23)		0.91 (0.52–1.62)		0.98 (0.75–1.28)	
50–54	Age 54→55	Ref.	<0.001	Ref.	0.120	Ref.	<0.001
	Age 53→55	0.84 (0.72–0.98)		0.88 (0.65–1.20)		0.83 (0.70–0.98)	
	Age 52→55	0.80 (0.70–0.91)		0.83 (0.64–1.09)		0.79 (0.68–0.92)	
	Age 51→55	0.79 (0.68–0.92)		0.77 (0.56–1.06)		0.80 (0.67–0.95)	
	Age 50→55	0.75 (0.65–0.86)		0.83 (0.63–1.09)		0.73 (0.63–0.86)	
55–59	Age 59→60	Ref.	<0.001	Ref.	0.022	Ref.	<0.001
	Age 58→60	0.88 (0.76–1.01)		0.88 (0.66–1.17)		0.88 (0.75–1.02)	
	Age 57→60	0.85 (0.73–0.98)		0.76 (0.55–1.06)		0.87 (0.73–1.03)	
	Age 56→60	0.78 (0.68–0.90)		0.79 (0.60–1.06)		0.78 (0.67–0.91)	
	Age 55→60	0.71 (0.60–0.83)		0.67 (0.48–0.94)		0.72 (0.60–0.86)	
60–64	Age 64→65	Ref.	<0.001	Ref.	0.001	Ref.	0.005
	Age 63→65	0.85 (0.75–0.97)		0.86 (0.66–1.12)		0.85 (0.74–0.99)	
	Age 62→65	0.89 (0.79–0.99)		0.80 (0.64–1.01)		0.91 (0.80–1.03)	
	Age 61→65	0.78 (0.68–0.89)		0.75 (0.57–0.99)		0.79 (0.67–0.91)	
	Age 60→65	0.82 (0.73–0.91)		0.68 (0.54–0.86)		0.86 (0.76–0.97)	
65–69	Age 69→70	Ref.	0.039	Ref.	0.150	Ref.	0.113
	Age 68→70	0.85 (0.75–0.97)		0.84 (0.61–1.14)		1.10 (0.94–1.30)	
	Age 67→70	0.89 (0.79–0.99)		0.78 (0.55–1.12)		1.00 (0.83–1.20)	
	Age 66→70	0.78 (0.68–0.89)		0.81 (0.60–1.10)		0.95 (0.80–1.12)	
	Age 65→70	0.82 (0.73–0.91)		0.73 (0.52–1.03)		0.92 (0.77–1.10)	
70–74	Age 74→75	Ref.	0.079	Ref.	0.149	Ref.	0.234
	Age 73→75	1.04 (0.90–1.20)		1.09 (0.75–1.60)		0.93 (0.75–1.15)	
	Age 72→75	0.95 (0.80–1.11)		1.05 (0.76–1.45)		0.97 (0.82–1.16)	
	Age 71→75	0.92 (0.79–1.06)		0.87 (0.59–1.26)		0.85 (0.70–1.03)	
	Age 70→75	0.88 (0.75–1.02)		0.85 (0.62–1.16)		0.89 (0.76–1.05)	
≥75	Age ≥79→age + 1 ^d^	Ref.	0.529	Ref.	0.437	Ref.	0.739
	Age 78→80	1.10 (0.88–1.38)		1.38 (0.84–2.25)		1.04 (0.81–1.34)	
	Age 77→80	0.91 (0.71–1.17)		1.01 (0.57–1.80)		0.89 (0.67–1.17)	
	Age 76→80	0.99 (0.81–1.20)		1.33 (0.87–2.03)		0.91 (0.73–1.14)	
	Age 75→80	0.96 (0.77–1.18)		0.65 (0.38–1.14)		1.03 (0.81–1.29)	

HR, hazard ratio; CI, confidence interval; ^a^ Participants within an age group were followed up until all reached comparable age. ^b^ Hazard ratios from multivariate model were adjusted for sex, obesity, smoking habit, alcohol consumption, physical activity, and comorbidity status. Regression model for total population was additionally adjusted for family history of gastric cancer status. ^c^ *p* for trend. ^d^ There was no age-limitation for age at *H. pylori* eradication; thus, for those who received *H. pylori* eradication at an age ≥79, the follow-up period was set to 1 year (age + 1). For example, people who received *H. pylori* eradication at the age of 82, the follow-up period was set to 1 year (up to the age of 83).

## Data Availability

The materials and data supporting the findings of the current study are available from the corresponding author upon reasonable request and IRB approval.
